# Physiological sensing for situational awareness: a theory-driven integrative review of multimodal and unsupervised approaches for visual search and human–autonomy teaming

**DOI:** 10.3389/fnrgo.2026.1797540

**Published:** 2026-04-29

**Authors:** Hicham Sekkati, Timothy Lam, Jean-Francois Lapointe, Luc Belliveau, Sebin Im

**Affiliations:** 1 National Research Council Canada, Ottawa, ON, Canada; 2 Defence Research and Development Canada, Toronto, ON, Canada

**Keywords:** EEG, human–autonomy teaming, multimodal fusion, physiological sensing, self-supervised learning, situational awareness, visual search

## Abstract

Situational awareness (SA) is fundamental to performance in visually demanding, safety-critical tasks and in human–autonomy teaming (HAT), yet existing measurement approaches remain limited. Direct probes such as SAGAT or SART disrupt task flow, while performance-based metrics capture outcomes rather than the underlying cognitive processes of perception, comprehension, and projection. This article presents a theory-driven integrative review of physiological sensing approaches for estimating SA. Physiological sensing provides a complementary, continuous, and objective alternative, with evidence that neural (EEG), ocular (eye tracking and pupillometry), and autonomic (ECG and EDA) signals each index distinct components of SA. The review synthesizes findings across these modalities with emphasis on two key domains: visual search tasks, where operators must detect, classify, and assess potential threats, and human–autonomy teaming, where effective coordination depends on shared SA, trust, and transparency. Beyond traditional feature-based pipelines, we examine recent advances in multimodal fusion and deep learning, highlighting the increasing role of unsupervised and self-supervised representation learning. These approaches exploit large volumes of unlabeled physiological data to reveal latent cross-modal structure, reduce reliance on sparse ground-truth labels, and enhance scalability and ecological validity. By integrating evidence across sensing modalities and computational frameworks, this review outlines the current state of physiological SA estimation and identifies key research directions for continuous, real-time monitoring in visual search and human–autonomy teaming.

## 1 Introduction

Situation-awareness (SA) refers to an individual's perception of environmental elements within a volume of time and space, the comprehension of their meaning, and the projection of their status into the near future (Endsley, 1995). Endsley's three–level model, *perception, comprehension*, and *projection*, has become the dominant theoretical framework across domains ranging from aviation and military operations to healthcare and human–robot interaction. Level 1 (perception) involves detecting and recognizing relevant cues in the environment; Level 2 (comprehension) entails integrating those cues to form an understanding of the current situation; and Level 3 (projection) concerns anticipating future states to guide decision making. SA is thus a dynamic, multi-stage cognitive construct that underpins effective performance where rapid and accurate interpretation of evolving information is critical.

SA remains a central component of effective performance in dynamic tasks such as aviation, surveillance, and driving. In visually demanding contexts, operators must rapidly detect potential targets, classify them as friends or enemies, and assess the threat level under conditions of uncertainty, distraction, and time pressure. Errors in any of these stages may compromise mission outcomes. Beyond individual operators, SA is equally critical in human–autonomy teaming (HAT), where humans and autonomous agents must develop a shared understanding of the environment, exchange intentions, and coordinate future actions. In such settings, operators must not only maintain their own awareness, but also anticipate autonomous behaviors and calibrate trust, as failures in shared SA can lead to misallocated attention, breakdowns in coordination, and degraded mission outcomes. Physiological sensing has therefore been explored as a way to estimate SA both for individual operators and, increasingly, for autonomous teammates. Such work aligns with neuroergonomics research showing that real-time neurophysiological measures (e.g., EEG engagement and workload indices) can track dynamic cognitive states during task performance (Berka et al., 2007), and may form the basis for adaptive human–machine systems that infer operator state and adjust information presentation or task demands. By providing real-time indicators of an operator's cognitive state, physiological monitoring enables the autonomous agent to adjust its behavior, such as modifying information flow, timing of alerts, or level of autonomy, in ways that help maintain shared SA and support the operator.

In parallel with the widespread adoption of Endsley's three-level framework, a substantial body of research has argued that SA is not solely an internal, individual cognitive product. Distributed cognition perspectives (Hutchins, 1995) conceptualize SA as a system-level property emerging from the interaction between people, tools, and environmental structures. From this view, perception and comprehension are shaped as much by external artifacts, interfaces, and coordination patterns as by internal mental models. Relatedly, team cognition frameworks emphasize that in collaborative or human–autonomy settings, SA is not simply the sum of individual awareness states but also the degree to which agents possess complementary, overlapping, or coordinated understandings relevant to joint goals (Cooke et al., 2013; Salas et al., 2015). Breakdowns in shared intent, communication, or mental-model alignment may thus degrade performance even when each individual operator maintains high individual-level SA. These perspectives do not replace Endsley's perceptual–comprehension– projection hierarchy but highlight its focus on the individual and its limits in multi-agent, tool-rich environments. They also motivate the need for SA assessment methods, including physiological sensing, that can capture not only internal cognitive dynamics but also interactions within distributed human–machine systems.

Traditional approaches to SA assessment include direct probes such as the Situation Awareness Global Assessment Technique (SAGAT), the Situation Awareness Rating Technique (SART), or the Situation Present Assessment Method (SPAM), and indirect performance measures such as accuracy, response time, and error rates. While informative, both approaches have well-documented limitations: probes provide only discrete snapshots and disrupt task flow, whereas performance metrics reflect end results rather than the evolving internal state of perception, comprehension, and projection. These constraints motivate the use of *physiological sensing*, which can capture continuous, objective markers of cognitive and affective processes without interfering with task execution. Recent studies demonstrate that electroencephalography (EEG) reveals transient neural markers of attention and target detection, eye tracking and pupillometry index gaze allocation and effort, and cardiovascular and electrodermal measures reflect workload and arousal. Together, these modalities offer complementary windows onto the three canonical levels of SA. However, analyzing them jointly is challenging: signals differ in sampling rates, noise characteristics, and temporal dynamics, and traditional feature-engineered classifiers often struggle to capture nonlinear interactions. Advances in deep learning provide a promising solution. Architectures such as convolutional and recurrent neural networks (CNNs and RNNs), transformers, and cross-modal attention mechanisms can learn spatiotemporal representations directly from minimally processed data while adaptively weighting modalities and time segments. Yet a persistent bottleneck remains: the scarcity of high-quality ground truth for SA. Direct probes yield sparse labels, and collecting them at scale in operational environments is costly and disruptive. These realities motivate the integration of *unsupervised and self-supervised learning*, which can exploit abundant unlabeled physiological data to discover latent multimodal representations and support downstream SA estimation with minimal labeled data. As learning-based approaches become increasingly central to SA estimation, questions of system behavior and interpretability take on added importance. From a cognitive engineering perspective, stable learning dynamics are critical for system transparency, predictability, and operator trust. Systems whose internal objectives evolve erratically may be difficult for human users to interpret, anticipate, or appropriately rely upon. By emphasizing analyzes of learning behavior, such as the interaction of distinct loss components during training, this review contributes to a clearer understanding of how complex learning systems converge and how their internal dynamics may influence downstream human decision making and system oversight.

The purpose of this review is therefore threefold and extends beyond descriptive synthesis. Importantly, physiological signals should not be interpreted merely as sensor measurements but as observable correlates of the cognitive mechanisms that support situation awareness. Neural signals such as EEG reflect rapid perceptual discrimination and attentional updating processes associated with Level-1 SA, whereas ocular metrics provide behavioral evidence of visual attention allocation during perceptual search and information sampling. Autonomic signals, including heart-rate variability and electrodermal responses, evolve over longer time scales and provide indirect indicators of cognitive workload, information integration, and anticipatory control processes that contribute to Level-2 and Level-3 SA. From this perspective, physiological sensing provides a bridge between cognitive theory and computational modeling: physiological signals capture the dynamics of perception, comprehension, and projection, while machine-learning models provide a mechanism for integrating these signals into continuous estimates of operator awareness.

**Figure 1:**
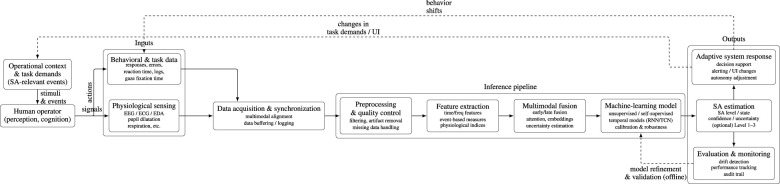
Conceptual framework for physiological sensing–based situation awareness estimation. Physiological and behavioral signals are processed through preprocessing, feature extraction, multimodal fusion, and machine-learning inference to estimate situation awareness with uncertainty. The framework also illustrates feedback loops in which adaptive system responses influence operator behavior and task context.

Beyond summarizing modality-specific findings, our aim is to bridge a gap in the literature by integrating physiological evidence with contemporary multimodal and self-supervised learning approaches. This synthesis provides a unified conceptual and computational framework for interpreting SA, distinguishing this review from prior surveys focused solely on physiological measures or machine-learning techniques. First, we synthesize findings on EEG, ocular, and autonomic measures relevant to SA in visually demanding tasks. Second, we extend this perspective to SA in human–autonomy teaming (HAT), where physiological sensing may support adaptive collaboration and trust calibration. Third, we highlight methodological trends, particularly the shift from feature-based pipelines to multimodal, unsupervised, and self-supervised learning approaches, and identify key challenges for developing continuous, interpretable, and real-time SA monitoring across both visual search and collaborative interaction settings. To clarify how physiological sensing, machine-learning inference, and adaptive system responses interact in operational environments, Figure 1 presents a conceptual framework for physiological situation-awareness estimation that links physiological sensing modalities to the cognitive architecture of SA. The framework illustrates how physiological signals reflecting perception, cognitive integration, and anticipatory processing are transformed through multimodal inference pipelines into operational estimates of situation awareness capable of supporting adaptive human–autonomy interaction. It also illustrates the end-to-end workflow linking physiological and behavioral signal acquisition to multimodal fusion, SA inference, and adaptive system responses.

To support a transparent and systematic review process, Section 2 describes the literature search and study selection procedure used in this review. The subsequent sections synthesize evidence on physiological sensing modalities, multimodal fusion approaches, and learning-based SA estimation methods, providing an integrated view of how physiological sensing, machine-learning models, and adaptive systems can support continuous situation-awareness monitoring in complex operational environments.

## 2 Literature search and selection methodology

The literature search and study selection followed a structured procedure inspired by PRISMA review guidelines (Page et al., 2021). The methodology describes the databases consulted, the search queries used and the inclusion and exclusion criteria applied to identify publications that address the use of physiological sensing to estimate situation awareness (SA), with particular attention to studies involving multimodal physiological signals and machine-learning approaches.

### 2.1 Search strategy

The literature search was conducted across several scientific databases commonly used in human factors, neuroscience, and engineering research, including IEEE Xplore, Scopus, Web of Science, and PubMed. Searches were performed between January 2025 and February 2026, retrieving publications from earlier years available in the selected databases. The search strategy combined keywords related to situation awareness, physiological sensing, and machine learning. Typical search queries included combinations of terms referring to situation awareness (e.g., “situation awareness” or “SA”), physiological monitoring (e.g., “physiological sensing” or “physiological monitoring”), and specific sensing modalities such as electroencephalography (EEG), eye tracking, heart-rate variability, electrodermal activity (EDA), and pupil dilation. Additional keywords referred to cognitive constructs closely related to situation awareness, including “cognitive workload” and “cognitive state,” as well as computational approaches such as “machine learning,” “deep learning,” and “multimodal fusion.” Boolean operators were used to combine these keywords in order to retrieve studies examining physiological indicators of cognitive processes relevant to SA, as well as research investigating computational methods for estimating such states.

To provide additional context on the evolution of research in this domain, a lightweight bibliometric inspection of the retrieved corpus was conducted. Publication metadata obtained from Scopus and Web of Science were used to examine the temporal distribution of studies and the prevalence of key research themes. The analysis indicated a clear growth in publications that address physiological sensing and machine-learning approaches to situation awareness after approximately 2016, coinciding with the broader adoption of multimodal sensing and deep-learning methods in neuroergonomics and human–machine interaction research. Keyword co-occurrence patterns further suggested that recent work increasingly combines physiological sensing modalities (e.g., EEG, eye tracking, and autonomic measures) with multimodal fusion and representation-learning approaches. This bibliometric overview was used only to contextualize research trends in the field; the detailed synthesis presented in this review focuses on representative and methodologically influential studies identified through the screening process described below.

### 2.2 Inclusion and exclusion criteria

Studies were considered eligible for inclusion if they were published in peer-reviewed journals or conference proceedings, written in English, and addressed situation awareness, cognitive-state estimation, or closely related constructs. Eligible studies also needed to incorporate physiological sensing modalities, such as EEG, ECG, electrodermal activity, eye tracking, or pupillometry. Both empirical studies and methodological contributions were considered, as well as review articles that provided relevant insights into physiological approaches to SA estimation. Studies were excluded if they did not involve physiological sensing or focused exclusively on behavioral performance measures without physiological indicators. Articles addressing cognitive constructs unrelated to situation awareness were also excluded. In addition, short abstracts, editorials, and non-peer-reviewed reports were not considered for inclusion in the review.

### 2.3 Screening and selection process

The initial search across databases yielded a broad corpus of publications. Titles and abstracts were first screened to identify studies potentially relevant to physiological sensing and situation awareness. In a second stage, full texts were examined to determine whether the studies met the inclusion criteria and contributed meaningful insights to the synthesis presented in this review. In addition to database searches, backward and forward citation tracking was used to identify additional relevant studies from the reference lists of key articles and recent review papers. This process helped ensure that foundational and highly cited works in the field were included.

### 2.4 Final corpus of reviewed literature

**Figure 2:**
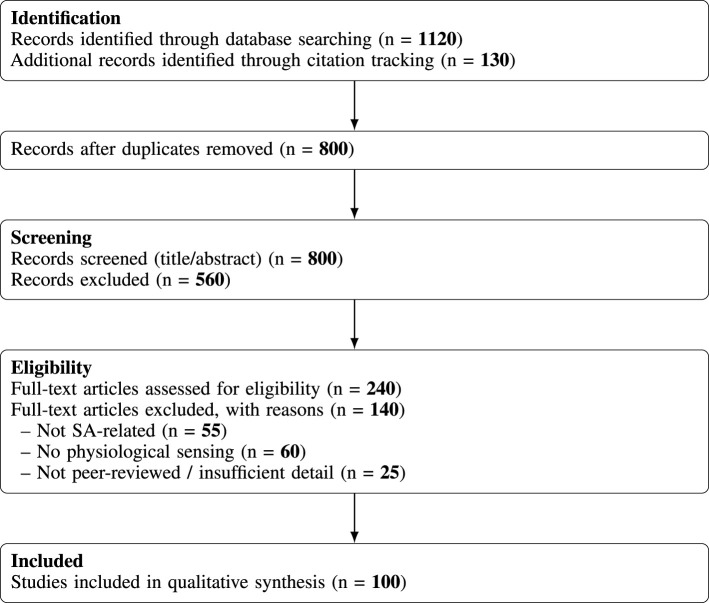
PRISMA-style flow diagram summarizing the literature identification, screening, eligibility assessment, and inclusion process.

Following the screening process, a subset of publications was retained for detailed analysis and synthesis. These studies collectively cover multiple physiological sensing modalities, experimental paradigms, and machine-learning approaches for estimating cognitive states associated with situation awareness. Rather than aiming for exhaustive coverage of all available publications, the goal of this review was to synthesize representative and influential studies across neuroergonomics, human factors, and computational modeling communities. The final corpus therefore reflects both methodological diversity and the most active research directions in the field. A schematic overview of the literature identification and screening process is illustrated in Figure 2.

## 3 Synthesizing evidence from systematic reviews of physiological measures of situation-awareness

Although the preceding section described how studies were identified, the present synthesis is organized around the cognitive structure of situation awareness rather than around sensing technologies alone. Specifically, the review interprets physiological signals in relation to the three cognitive levels of SA: perception, comprehension, and projection. This theoretical lens enables comparison of studies employing different sensing modalities, task paradigms, or machine-learning models by linking them to the underlying cognitive mechanisms they aim to measure. By structuring the synthesis around these cognitive processes, the review moves beyond a purely descriptive catalog of sensing technologies and instead provides a conceptual interpretation of how physiological indicators relate to the dynamic formation of situation awareness. The studies identified through the search procedure were synthesized using a *theory-driven integrative review* approach that emphasizes conceptual and methodological insights rather than quantitative aggregation of individual effect sizes. This article does not present new experimental data; instead, it integrates and interprets findings reported in previously published studies to provide a structured overview of physiological sensing approaches to situation-awareness estimation. The objective is to integrate evidence across three intersecting research areas: (i) physiological correlates of situation awareness, (ii) multimodal fusion and machine-learning approaches for cognitive-state estimation, and (iii) emerging unsupervised and self-supervised representation-learning frameworks. To support this synthesis, the analysis draws primarily on recent systematic reviews and methodological surveys in human factors, cognitive engineering, neuroscience, and biomedical signal processing. By reorganizing these findings within a unified conceptual framework, the review highlights cross-modal regularities, identifies methodological gaps that persist across application domains, and connects advances in learning-based modeling with broader cognitive engineering concerns such as interpretability, trust, and decision support. In this way, the article complements prior systematic efforts by translating accumulated empirical evidence into design-relevant insights and future research directions for cognitive engineering and decision-making systems.

To situate our contribution within the broader research landscape, we conducted a detailed review of prior work examining physiological measures in relation to SA. This review synthesizes studies that have explored diverse modalities, ranging from neural signals (e.g., EEG/ERPs) to peripheral indicators such as heart rate, electrodermal activity, and respiration, with the goal of assessing how these signals inform perception, comprehension, and projection. By systematically organizing existing findings, we highlight both the demonstrated potential of these modalities and the limitations of current approaches. These insights motivate the structured mapping presented in the next section and clarify the need for an integrative perspective on multimodal fusion. Several systematic reviews have evaluated the state of physiological sensing for SA. The most comprehensive effort to date is provided in (Zhang et al. 2023), which reports a systematic review that initially screened 1,178 records and ultimately synthesized 25 primary studies spanning healthcare, construction, transportation/driving, process control and other critical safety domains. Their review identified three consistent themes: (1) eye–movement metrics such as fixation patterns, dwell time, and gaze allocation showed the most reliable associations with direct SA probes such as SAGAT and SART; (2) EEG indices, including absolute alpha before task resting, alpha frontal asymmetry, and band power ratios, provided promising but still limited evidence due to the small number of studies and methodological heterogeneity; and (3) peripheral measures such as heart rate variability (HRV) and electrodermal activity (EDA) showed sensitivity to workload and stress but produced mixed results regarding their specificity for SA.

Other targeted reviews converge on these observations. For example, the findings reported in (Mahanama et al. 2022) show that the ocular metrics consistently track the perception and comprehension levels of SA in dynamic visual tasks, whereas the cardiovascular responses capture slower global workload fluctuations more closely aligned with projection or anticipation. The evidence summarized in (Baldwin et al. 2017) indicates that individual physiological or behavioral modalities rarely achieve high predictive power for cognitive state or SA compared to direct ground truth, and that multimodal fusion offers superior robustness and precision. A methodological review of heart rate variability metrics (Shaffer and Ginsberg, 2017) highlights how context, recording protocols, and physiological confounds affect interpretability. A broader systematic synthesis (Zhang et al., 2023) notes that only a minority of studies combine physiological signals with validated SA measures such as SAGAT or SPAM, limiting interpretability and cross-study comparability. Sample sizes were often small, sometimes fewer than 20 participants, hindering statistical power and the feasibility of data-hungry machine learning approaches. Task paradigms also varied widely (e.g., simulated flight, driving, healthcare, construction, and process control), introducing domain-specific confounds and preventing standardized feature extraction.

Despite these limitations, several cross-cutting opportunities emerge. Eye tracking, and in some cases pupillometry, represent some of the most validated behavioral and physiological signals for assessing Level-1 and Level-2 situation awareness in domains such as aviation, surveillance, human–robot interaction, and vehicle operation (Zhang et al., 2023; Baldwin et al., 2017). These measures have demonstrated reliable correlations with SA in simulator and training environments, although their deployment in field settings and their consistency across operational contexts remain areas of active research. Electroencephalography (EEG), including event-related potentials and spectral metrics, provides unique temporal precision and direct access to perceptual decision processes, although its practical use requires careful artifact rejection and often substantial numbers of within-subject trials to obtain reliable estimates (Hogervorst et al., 2014; Festa et al., 2025). Peripheral autonomic measures, such as heart rate and heart-rate variability (HR/HRV), electrodermal activity (EDA), and respiration, offer robust indicators of arousal and workload and are relatively easy to collect in operational environments. However, these signals are generally most informative when interpreted in combination with central neural or ocular measures (Shaffer and Ginsberg, 2017).

The present visual' search–focused review follows this trajectory by linking modality' specific evidence to the three-level SA model. Taken together, these systematic reviews reveal both the promise and fragmentation of current physiological approaches to SA. Each modality captures a distinct aspect of perception, comprehension, or projection, yet none alone provides a complete representation of the operator's evolving awareness state. This fragmentation has spurred increasing interest in multimodal integration, leveraging complementary signals to improve sensitivity, specificity, and robustness. Moreover, advances in machine learning, particularly deep learning, now enable automatic discovery of cross-modal patterns that traditional feature-based pipelines could not capture. The following section therefore examines how multimodal fusion and modern learning architectures have been applied to unify these disparate physiological indicators into coherent, data-driven frameworks for SA estimation.

From a cognitive engineering perspective, the value of physiological situation-awareness (SA) estimation lies not in classification accuracy *per se*, but in how inferred SA states shape downstream *decision making, interface adaptation*, and *human–autonomy coordination*. Physiological indicators provide probabilistic, time-varying estimates of perceptual, integrative, and anticipatory processes that directly influence operators' ability to select, time, and execute decisions under uncertainty. When integrated into decision-support systems, SA estimates can inform *adaptive interface strategies* that align information presentation with the operator's current cognitive state. For example, indicators of degraded perceptual SA (e.g., attentional tunneling reflected in gaze concentration or reduced P300 responses) can trigger visual reorganization, cue highlighting, or multimodal alerts, whereas markers of high cognitive load or reduced comprehension (e.g., sustained frontal theta or reduced HRV) may motivate information throttling, temporal deferral of noncritical messages, or automation of low-level tasks. At the projection level, anticipatory physiological signatures can support predictive adaptation, such as early intervention before loss of vigilance or delayed response to future task demands.

Crucially, physiological SA estimation introduces new *failure modes* that must be explicitly addressed in safety-critical systems. False positives, incorrectly inferring low SA, may lead to unnecessary automation intrusions, alert fatigue, or erosion of operator trust. False negatives, by contrast, risk missed interventions during genuine awareness breakdowns. Additional failure modes arise from sensor degradation, individual differences, contextual confounds (e.g., stress unrelated to task demands), and model overfitting to laboratory conditions. These risks underscore the need to treat physiological SA estimates as *decision-support signals* rather than ground truth, and to couple them with uncertainty quantification, redundancy across modalities, and conservative adaptation policies. From a design standpoint, effective integration therefore requires that (1) SA estimates be communicated to the autonomy and interface layers in probabilistic or confidence-weighted form; (2) adaptations be graded and reversible rather than binary; and (3) operators retain clear visibility into why and when system behavior changes. Interpretable models and transparent adaptation logic support mental-model alignment, enabling operators to anticipate system responses and calibrate trust appropriately. In this way, physiological SA estimation becomes a mechanism not only for detecting cognitive state, but for shaping human–autonomy interaction in a manner that preserves agency, supports robust decision making, and mitigates cascading failures in complex sociotechnical systems.

### 3.1 Analytical dimensions for comparing physiological SA measures

To address the need for deeper analytical structuring, the reviewed studies can be organized along a set of complementary dimensions that capture how physiological signals contribute to situation awareness (SA). These dimensions define measurable variables, such as signal latency, level of derivation, and temporal integration window, which enable systematic comparison across modalities. A first important distinction concerns the temporal scale of the signal. Physiological modalities differ substantially in their temporal resolution and latency: EEG and ocular signals operate at millisecond scales and are therefore well suited to capturing rapid perceptual processes associated with Level 1 SA, whereas cardiovascular and electrodermal signals evolve over longer time scales and are more informative for sustained cognitive integration and anticipatory processes related to Levels 2 and 3. A second dimension relates to observability and processing requirements. Some indicators are directly observable, such as fixation location or heart rate, while others require event-based or post hoc processing, such as ERP components like the P300. This distinction is critical for real-time applicability, as signals that depend on stimulus alignment or trial averaging are less suitable for continuous SA estimation. Another key dimension concerns the specificity of physiological signals to SA levels. Ocular metrics tend to show relatively direct associations with perceptual attention, whereas autonomic signals often reflect broader constructs such as workload or arousal, requiring contextual interpretation to infer their relationship to comprehension or projection. In addition, modalities differ in their robustness and ecological validity. Neural signals such as EEG provide rich information about cognitive processes but are sensitive to motion artifacts and environmental noise, whereas cardiovascular signals are generally more robust but less specific to particular cognitive mechanisms. Finally, modalities vary in their suitability for real-time use. Eye tracking and heart rate can be computed continuously with low latency, while ERP-based measures typically require event alignment and averaging, limiting their direct applicability in operational settings. Taken together, these dimensions reveal consistent patterns across studies; fast and directly observable signals dominate perception-level SA, whereas slower and more integrated physiological responses contribute to comprehension and projection. This structured perspective provides a foundation for the operational and computational framework introduced in the following sections.

## 4 Multimodal fusion and machine learning

As summarized in Section 3, physiological measures rarely provide a complete picture of SA when considered in isolation. EEG may index rapid perceptual or decision related processing, eye tracking provides spatial attention markers, and cardiovascular or electrodermal activity reflects slower autonomic arousal. Because these signals capture partly independent aspects of the perception–comprehension–projection framework, combining them can improve both sensitivity and specificity in predicting moment to moment SA states (Hogervorst et al., 2014; Baldwin et al., 2017; Wilson and Russell, 2003a).

In HAT scenarios, maintaining operator SA becomes increasingly challenging as responsibility and information flow are distributed between humans and autonomous agents. Physiological sensing offers a means of inferring the operator's moment-to-moment cognitive state, and fusion models can combine multiple physiological channels, such as eye movements, EEG, or cardiovascular measures, to improve the robustness of individual SA estimation. Our focus in this review is therefore on operator-centric fusion approaches. However, the same fusion principles could be extended in future work to incorporate additional contextual signals, such as system-state information from autonomous teammates. Such an extension would enable more holistic joint models capable of estimating shared or team-level SA, though this lies beyond the scope of the present review. Recent systematic reviews consistently identify two major methodological trajectories in SA estimation: (1) the rise of multimodal physiological sensing combined with traditional machine learning, and (2) the growing use of deep learning for automatic representation learning from raw or minimally processed signals. These trends mark a clear evolution from single-channel, theory-driven metrics toward multimodal, data-driven models capable of estimating SA continuously and in real time. A central motivation for multimodal physiological sensing is that cognitive states relevant to SA and related constructs (e.g., workload) are underdetermined by any single signal, but can be more robustly inferred by combining complementary neural, ocular, and autonomic measures that each reflect partially overlapping aspects of cognitive control, engagement, and arousal (Hogervorst et al., 2014).

### 4.1 Classical fusion strategies: combining partial views

Early work typically followed a two-stage pipeline for estimating SA from physiological data. In these approaches, researchers first extracted handcrafted features from individual signal modalities, for example, fixation duration and saccade rates from eye tracking, frequency-band power from EEG, or HRV metrics from cardiac data. A separate machine-learning classifier, such as an SVM, k-NN, or decision tree, was then trained to map these engineered features to predefined SA levels. Within the framework adopted in this review, such early methods correspond to a manually specified feature-extraction stage feeding into a supervised fusion model, illustrating the lower end of the fusion spectrum where feature construction is explicitly designed rather than learned. Fusion in these systems is commonly performed either at the feature level, where the engineered features of each modality are concatenated into a single representation, or at the decision level, by combining posterior probabilities or votes from individual modality-specific classifiers (Hogervorst et al., 2014; Yang et al., 2023; Jia et al., 2024; Qian et al., 2024a; Smith et al., 2025). Feature-level fusion offers compactness and simpler training pipelines, but can suffer from misaligned temporal resolutions and sensitivity to noisy modalities. Decision-level fusion, by contrast, improves robustness to modality failures and enables modality-specific modeling, but can incur higher computational cost when posterior probabilities are derived via sampling-based methods (e.g., MCMC) rather than gradient-based optimization. Despite these trade-offs, both strategies remain attractive because they are interpretable, require smaller training sets, and allow an explicit examination of each modality's contribution to the SA estimation. Recent surveys further expand these taxonomies, providing a more complete description of multimodal fusion strategies for biomedical signals (Stahlschmidt et al., 2022).

### 4.2 Deep learning architectures for multimodal situation-awareness estimation

More recently, deep learning methods have begun to reshape multimodal SA prediction. Convolutional neural networks (CNNs) and recurrent neural networks (RNNs), including long short-term memory (LSTM) and gated recurrent unit (GRU) models, can learn spatiotemporal patterns directly from raw or minimally processed signals, thereby reducing the need for handcrafted features. CNNs operate by applying convolutional kernels that detect spatially localized patterns, while LSTM and GRU architectures specialize in modeling temporal dynamics through gated memory cells that preserve or update information across time steps (Schirrmeister et al., 2017; Bashivan et al., 2015; Chen et al., 2024a; Xu et al., 2025).

Multimodal architectures integrate heterogeneous inputs through several complementary strategies. Early fusion CNN/RNN models jointly learn cross-modal representations from synchronized time–frequency maps, such as EEG spectrograms combined with pupil diameter or heart-rate time series, and have demonstrated improved detection of SA-related fluctuations in realistic surveillance and monitoring tasks (Zheng et al., 2019; Yin et al., 2021). Attention mechanisms and transformer-based architectures further enhance multimodal modeling by allowing networks to adaptively weight different modalities and temporal segments, highlighting the most informative channels for each prediction window (Vaswani et al., 2017; Xu et al., 2025). In addition, graph-based and attention-driven neural networks, such as bi-hemispheric discrepancy RNNs (Li et al., 2021) and 3D convolution–attention models (Liu et al., 2022), illustrate the capacity of deep architectures to capture complex spatiotemporal dependencies and regional asymmetries across EEG channels. Although these approaches currently operate primarily within a single modality, their graph-inspired and attention-based structures provide a natural foundation for future multimodal extensions that integrate heterogeneous physiological sensors.

In summary, recent work illustrates a clear methodological progression, from single-channel, theory-driven analyses to multimodal, data-driven models capable of continuously estimating SA in real time (Chen et al., 2024b). Deep learning architectures such as convolutional and recurrent neural networks have demonstrated strong performance in related domains including workload detection, fatigue monitoring, and affect recognition, and emerging studies report comparable gains for SA estimation in dynamic and high-demand tasks (Jia et al., 2024; Smith et al., 2025). Nevertheless, these advances remain constrained by the scarcity of high-quality labeled data and the difficulty of obtaining continuous ground truth for SA without interrupting task performance. This limitation becomes even more pronounced in HAT contexts, where the concept of shared or distributed SA requires aligning physiological and behavioral indicators from the human operator with system-state information from the autonomous teammate. Such synchronization and labeling are inherently challenging, making it difficult to obtain reliable annotations that reflect team-level SA rather than individual perspectives.

### 4.3 Toward unsupervised and self-supervised modeling

As emphasized by recent work (Zong et al., 2023), these challenges have prompted growing interest in unsupervised and self-supervised learning (SSL) methods, which can leverage large volumes of unlabeled physiological and behavioral data to uncover latent, cross-modal representations. A number of public multimodal physiological datasets have become de facto benchmarks for affect-, workload-, and vigilance-related tasks in SA-adjacent research. Representative examples include DEAP (EEG + peripheral physiology), MAHNOB-HCI (EEG, ECG, eye tracking, video), AMIGOS (EEG, ECG, GSR, video), DREAMER (EEG + ECG), and WESAD (wearable chest/wrist ECG, EDA, BVP, respiration, accelerometry). Although these corpora provide valuable testbeds for supervised and multimodal fusion approaches, they remain limited in subject count, rely on laboratory elicitation protocols, and rarely include validated SA probes—constraints that directly reinforce the broader “label scarcity” problem in SA modeling. Supervised pipelines remain the dominant approach on these datasets, yet they are prone to overfitting when annotated data are sparse. Semi-supervised and transfer-learning strategies mitigate label requirements by reusing labeled corpora (e.g., pre-training on one dataset and fine-tuning on another), though their effectiveness depends heavily on domain similarity and consistent preprocessing. In contrast, unsupervised and self-supervised pre-training methods, including autoencoders, variational autoencoders (VAE), contrastive representation learning, masked-signal modeling, and predictive-coding objectives, have shown the ability to learn robust, reusable physiological representations from large collections of unlabeled data. These methods can discover low-dimensional latent structures shared across modalities without explicit SA labels, potentially capturing cognitive factors (e.g., vigilance, comprehension load, and projection-related anticipatory dynamics) that are otherwise difficult to annotate directly. Recent reviews highlight the growing importance of multimodal fusion and representation-learning frameworks, particularly unsupervised and self-supervised approaches, for biomedical and neurophysiological signals, which enable cross-modal feature discovery and reduce dependence on extensive labeled datasets (Stahlschmidt et al., 2022). Recent empirical work further demonstrates these benefits. Modality-agnostic transformer frameworks such as MATS2L (Goetz et al., 2023) extract shared representations across heterogeneous physiological channels; multiscale temporal contrastive predictive coding has shown strong performance on EEG time series in low-label regimes (Xu et al., 2023); and cross-subject contrastive learning approaches improve transferability across individuals and sessions in motor-imagery EEG (Li et al., 2024). Several of these studies report state-of-the-art improvements when applying SSL pretraining to DEAP-, WESAD-, or DREAMER-style benchmarks, reinforcing the potential of these techniques for SA modeling. Together, these findings indicate that SSL methods can substantially reduce reliance on high-quality SA labels, support cross-subject generalization, and motivate the development of standardized physiological SSL benchmarks tailored to SA research.

Unsupervised modeling is particularly relevant for SA estimation in operational settings where ground-truth probes may disrupt ongoing tasks and compromise safety, where data distributions shift across operators, equipment, or mission phases, and where privacy constraints limit the availability of detailed behavioral labels. By capturing structure in the data itself, unsupervised and self-supervised frameworks can facilitate cross subject transfer, enable anomaly detection for rare events, and reduce the need for costly manual annotation. Hybrid pipelines that combine unsupervised feature extraction with lightweight supervised fine tuning (e.g., few90shot learning or semi90supervised calibration) are therefore a natural next step toward real-time, deployable SA monitoring.

### 4.4 Current challenges and opportunities

Despite encouraging progress, several limitations persist in the development of multimodal physiological models for SA estimation. Below we expand the major challenges and outline actionable research directions for addressing them.

#### 4.4.1 Data scarcity and limited ecological diversity

Most SA datasets contain fewer than 30 participants, include short recordings, and rely on controlled laboratory paradigms, restricting model generalization (Hogervorst et al., 2014; Stahlschmidt et al., 2022). Remedies include (1) label-efficient self-supervised pre-training (Section 4), which has improved downstream performance in low-label regimes in several recent physiological benchmarks (Goetz et al., 2023; Xu et al., 2023; Li et al., 2024); (2) cross-dataset pretraining and domain adaptation to mitigate distribution shifts across tasks and physiological conditions (Ganin et al., 2016; Wang et al., 2024); and (3) synthetic augmentation using generative or physiologically informed models, which can expand the space of plausible signals and have shown benefits in some EEG applications, although careful validation is essential to ensure physiological fidelity and avoid artifacts (Hartmann et al., 2018; Neifar et al., 2025). An important longer-term opportunity is the creation of large-scale multimodal SA benchmarks with standardized protocols and shared metadata (Delorme, 2023).

#### 4.4.2 Lack of standardization across tasks, sensors, and labeling protocols

Heterogeneity in preprocessing pipelines, sensor configurations, sampling rates, and SA ground-truth definitions complicates cross-study comparison (Zhang et al., 2023). Actionable solutions include (1) unified preprocessing toolkits and reproducible pipelines for EEG, ocular, and autonomic signals (Pedroni et al., 2019; Delorme, 2023); (2) dataset releases enriched with contextual metadata about task structure, sensor placement, and labeling (Koelstra et al., 2012; Healey and Picard, 2003); and (3) community benchmarks with common evaluation metrics for continuous SA estimation. Standardized ontologies for SA labels, aligned with perception, comprehension, and projection, would further improve reproducibility and interoperability (Endsley, 1995).

#### 4.4.3 Interpretable modeling for safety-critical decision support

Deep and unsupervised models enable single-trial prediction and real-time adaptation, but they introduce challenges related to transparency, regularization, and stability (Samek et al., 2017). For SA-related visual search and target-detection tasks, existing evidence suggests that the most effective architectures are those that balance spatiotemporal representation learning with interpretability, namely: (1) convolutional and recurrent networks for structured temporal signals such as EEG and ocular dynamics; (2) transformer-based multimodal encoders when heterogeneous modalities must be integrated; and (3) self-supervised variants of these models that reduce label requirements while preserving discriminative structure. These architectures have been repeatedly adopted in recent physiological–cognitive-state studies (Goetz et al., 2023; Xu et al., 2023; Li et al., 2024), whereas physics-informed neural networks (PINNs) are less commonly applied to target detection because they require explicit governing equations—constraints that are difficult to formalize for cognitive processes such as attention allocation or visual SA. Nevertheless, PINNs remain valuable for interpretable latent-state inference in physiological systems governed by physical dynamics (Raissi et al., 2019; Karniadakis et al., 2021; Ahmadi et al., 2025; Willard et al., 2022). Hybrid strategies therefore offer a practical compromise: using deep encoders optimized for target detection in conjunction with interpretable shallow classifiers or *post-hoc* explanations such as attention visualization and Shapley-value attribution (Lundberg and Lee, 2017). When the embedded dynamics faithfully reflect the underlying physiology, such approaches can yield models that are both performant and mechanistically interpretable (Rudy et al., 2017; Luo et al., 2025).

#### 4.4.4 Interpretability, learning dynamics, and operator mental models

Whereas the preceding paragraph focuses on architectural and methodological approaches to interpretability, this section shifts the emphasis to how learning dynamics and latent representations are understood by human operators and how they align with cognitive models of situation awareness. In safety-critical cognitive systems, interpretability is not solely a property of model architecture but a function of how learning dynamics align with operators' mental models of perception, comprehension, and projection. From a situation-awareness (SA) perspective, interpretable representations are valuable to the extent that they correspond to cognitively meaningful stages of information processing and evolve in predictable ways over time. Learning systems whose internal objectives or dominant loss components fluctuate erratically during training or adaptation risk producing outputs that are difficult for human operators to anticipate, interpret, or trust. Interpretable multimodal representations can be explicitly mapped onto the three canonical levels of SA. At the *perception* level, models that expose spatial or temporal attention weights (e.g., saliency over visual regions, EEG channels, or time windows) align with operators' intuitive understanding of cue detection and attentional allocation. For example, an attention map that emphasizes peripheral gaze neglect during a visual search task provides a cognitively legible explanation for a detected loss of perceptual SA. At the *comprehension* level, latent states reflecting sustained working-memory load, cross-modal integration, or conflict monitoring correspond to the operator's internal effort to construct meaning from multiple information sources. At the *projection* level, slowly evolving latent variables associated with anticipatory control or uncertainty tracking can be interpreted as indicators of the operator's ability to foresee future task states and plan accordingly. Learning dynamics play a critical role in sustaining this interpretability. When distinct objectives, such as reconstruction loss, contrastive alignment, and supervised calibration, are combined, their relative influence over training time shapes which cognitive processes are emphasized in the learned representation. Stable convergence patterns, in which early learning prioritizes perceptual structure and later stages refine integrative and predictive features, are more consistent with human notions of progressive SA formation. In contrast, unstable or oscillatory dominance between loss terms may yield representations that shift unpredictably between perceptual and higher-level abstractions, undermining transparency and complicating trust calibration. Consider a hypothetical adaptive UAV interface that relies on a multimodal, self-supervised SA estimator. If the model consistently attributes low SA to reduced visual scanning (perception-level deficit), the interface may adapt by highlighting missed regions or increasing visual salience. If, however, the same model intermittently attributes low SA to anticipatory failure (projection-level deficit) without clear contextual cues, operators may perceive the system's interventions as arbitrary or misaligned with their own experience. In this case, interpretability is not merely about exposing internal variables, but about ensuring that model inferences correspond to plausible cognitive explanations from the operator's perspective.

These considerations motivate hybrid modeling strategies that combine expressive representation learning with cognitively grounded constraints. Examples include architectures that enforce temporal separation between perceptual and predictive latent states, hierarchical losses aligned with SA levels, or *post-hoc* explanations that explicitly reference perception, comprehension, or projection. Such approaches support the development of systems whose learning behavior remains intelligible throughout training and deployment, thereby enhancing transparency, predictability, and trust in human–autonomy teaming contexts.

#### 4.4.5 Data security, privacy, and cross-institutional scalability

Physiological and behavioral signals contain sensitive cognitive and health-related information. Beyond encryption and access controls, privacy-preserving ML frameworks are required to enable large-scale multimodal SA research. Federated learning allows distributed model training without sharing raw data (McMahan et al., 2017), supporting multi-institution collaboration in domains such as medical imaging (Sheller et al., 2020). Extending federated approaches to scenarios where institutions contribute heterogeneous modalities remains an active research direction. Complementary techniques such as differential privacy, secure aggregation, and homomorphic encryption can further protect gradients and model updates during training (Truex et al., 2019). These frameworks collectively point toward viable pathways for privacy-preserving, cross-institutional SA modeling at scale.

#### 4.4.6 Inter-individual variability and cross-subject transfer

Substantial variability in physiological responses across individuals, equipment setups, and operational conditions limits model generalization (Wang et al., 2023). Remedies include subject-invariant representation learning, adversarial alignment (Ganin et al., 2016), and calibration-light personalization via few-shot or semi-supervised adaptation (Zhao et al., 2020). Transformer-based and graph-structured multimodal architectures offer additional opportunities by encoding individual differences through subject embeddings or context-conditioned normalization.

In summary, while deep and self-supervised models enable single-trial prediction and real-time adaptation, meaningful opportunities remain to advance standardization, interpretability, privacy, and cross-institution scalability. Addressing these challenges will be crucial for transitioning multimodal physiological SA estimation from controlled laboratory paradigms to robust, transparent, and ethically deployable systems in real-world human–autonomy teaming.

### 4.5 Synthesis and implications for human–autonomy teaming

Multimodal fusion offers a principled framework for estimating, monitoring, and enhancing situation-awareness in human–autonomy teaming (HAT). Across the reviewed literature, a convergent pattern emerges: SA is best predicted not by any single channel but by the synergistic interaction between physiological, behavioral, and system-state information. This observation aligns with operational demands in contemporary HAT settings, where operators must interpret complex environments while coordinating with autonomous teammates.

#### 4.5.1 Operational relevance in automated driving and UAV control

In partially automated driving, multimodal fusion models can combine ocular metrics (reflecting visual attention), EEG indicators of vigilance, and vehicle-state telemetry to anticipate lapses in monitoring or overtrust in automation. Such systems can trigger adaptive interfaces, e.g., increasing auditory alerts during low-SA states, or modulate the autonomy level when the operator becomes overloaded. Similarly, in UAV operations, fusing EEG, heart-rate variability, eye tracking, and mission-state variables enables real-time detection of attentional tunneling, loss of spatial awareness, or difficulty integrating sensor feeds. Autonomous agents can then modify information flow, prioritize mission cues, or redistribute tasks across the human–machine team. These examples illustrate that multimodal fusion is not only a methodological advancement but a practical mechanism for maintaining shared SA in real-world HAT environments.

#### 4.5.2 Connecting the machine-learning subsections

Although the preceding subsections addressed supervised learning, self-supervised fusion, and interpretable modeling separately, together they illustrate a coherent progression. Supervised models provide strong performance when high-quality labels exist but exhibit limited transferability. Unsupervised and self-supervised methods alleviate label scarcity and enable cross-dataset pre-training, thereby improving generalization. Interpretable and physics-informed architectures then address safety and transparency requirements in high-consequence HAT domains. This progression, from accuracy, to scalability, to interpretability, highlights the layered nature of modern SA modeling: no single approach is sufficient, but the combination forms an integrated pipeline from data acquisition to real-time decision support.

#### 4.5.3 Toward integrated fusion frameworks

A unified future direction is the development of end-to-end multimodal fusion models that incorporate the strengths of each approach: supervised fine-tuning for mission specificity, self-supervised representation learning for label efficiency, and mechanistic constraints for interpretability. Practical deployment will also require embedded uncertainty quantification, calibration-light personalization, and privacy-preserving learning strategies to support scalable training across institutions.

#### 4.5.4 Implications for future HAT systems

When implemented in operational settings, these multimodal fusion approaches offer three key benefits: (1) real-time estimation of operator and team-level SA, enabling adaptive autonomy; (2) increased robustness to environmental variability and individual differences; and (3) improved transparency and trust calibration through interpretable feedback. These capabilities directly address emerging challenges in autonomous driving, UAV swarm operations, and distributed command-and-control systems, where maintaining shared SA is crucial for safety and mission success.

## 5 Operationalizing situation awareness from physiological signals

Although physiological sensing provides rich information about cognitive processes related to situation awareness (SA), translating these signals into measurable and operational indicators remains a key challenge. To address this limitation, we introduce an operational framework that links physiological features to SA levels through measurable variables, temporal constraints, and decision logic suitable for real-time estimation.

### 5.1 Measurable indicators of SA levels

Physiological signals must be expressed through observable or computable features that can be extracted continuously. For perception (Level 1 SA), rapid neural and ocular responses provide primary indicators. For example, EEG spectral features (e.g., alpha suppression) and event-related responses (e.g., P300) reflect attentional engagement with salient stimuli, while eye-tracking metrics such as fixation probability, time-to-first-fixation, and gaze concentration over task-relevant regions provide direct evidence of perceptual allocation. For comprehension (Level 2 SA), indicators reflect sustained cognitive integration. These include frontal theta activity in EEG, structured scanpath patterns in eye tracking, and reductions in heart-rate variability (HRV), which are associated with increased cognitive workload and information processing demands. Projection (Level 3 SA) can be operationalized through anticipatory and predictive physiological dynamics. These include temporal modulation of frontal theta activity, anticipatory changes in HRV prior to expected events, and electrodermal responses preceding critical task transitions. Unlike perception, projection requires integrating temporal context and is therefore inherently dependent on longer observation windows and predictive modeling.

### 5.2 Operational thresholds and decision logic

To enable practical use, physiological indicators must be associated with thresholds or decision rules. For example, perceptual SA may be inferred when fixation probability on task-relevant regions exceeds a defined threshold within a given time window, combined with evidence of neural engagement (e.g., alpha suppression). Similarly, comprehension-level SA may be associated with sustained frontal theta activity and stable scanpath organization over intermediate temporal windows. Rather than relying on fixed thresholds, operational SA estimation can be formulated probabilistically. Multimodal features can be combined to estimate the likelihood of each SA level:


$$\begin{array}{l} P\left(SA_{t}|{\text{x}}_{t}\right)=f\left({\text{x}}_{EEG},{\text{x}}_{eye},{\text{x}}_{auto}\right) \end{array}$$ (1)

where **x**_*t*_ represents multimodal physiological features within a temporal window.

### 5.3 Real-time constraints and signal selection

A critical consideration for operational SA estimation is real-time feasibility. Not all physiological measures are equally suitable for continuous monitoring. Event-related potentials (ERPs), such as the P300, require stimulus alignment and often trial averaging, limiting their direct applicability in real-time systems. In contrast, spectral EEG features, gaze metrics, and heart-rate measures can be computed continuously with low latency and are therefore more appropriate for online SA estimation. This distinction motivates the use of hybrid approaches in which event-based neural markers are used for offline validation or calibration, while continuous features support real-time inference. Sliding-window analysis provides a practical solution, with different temporal windows corresponding to different SA levels (e.g., short windows for perception, longer windows for comprehension and projection).

### 5.4 A hierarchical and predictive model of situation-awareness dynamics

Rather than relying solely on fixed thresholds, operational SA estimation can be formulated probabilistically, allowing uncertainty to be explicitly modeled. Multimodal features can be combined to estimate the likelihood of each SA level; Perception (Level 1) provides input for comprehension (Level 2), which in turn supports projection (Level 3). Physiological responses therefore evolve across stages, reflecting a progression from rapid detection to integration and anticipation. Fast and directly observable signals, such as gaze allocation and EEG spectral responses, primarily reflect early perceptual processes. These signals provide the basis for subsequent integration, where slower and more sustained physiological dynamics, such as frontal theta activity and heart-rate variability, reflect comprehension-level processing. Projection, in turn, emerges from the temporal integration of these signals, incorporating anticipatory dynamics and predictive control mechanisms that depend on longer observation windows. This hierarchical relationship can be expressed as a temporal process:


$$\begin{array}{l} SA_{t}=f\left(L1_{t},L2_{t},L3_{t}\right) \end{array}$$ (2)

where each level is estimated from modality-specific features and their temporal evolution. Projection, in particular, requires predictive modeling. Machine-learning models such as recurrent neural networks or temporal transformers can capture dependencies over time and infer future-oriented cognitive states from past physiological dynamics. This enables estimation not only of current SA but also of its likely evolution. This hierarchical and dynamic perspective clarifies that physiological modalities do not map exclusively to single SA levels, but contribute differently across stages of SA formation. It also provides a structured basis for comparing results between studies, as differences can often be explained by variations in temporal scale, task demands, and the stage of SA being measured.

### 5.5 Illustrative application

In a UAV surveillance scenario, reduced fixation on target regions combined with weak neural engagement may indicate degraded perception-level SA, triggering visual cue enhancement. Sustained high cognitive load reflected in EEG and HRV may indicate comprehension difficulties, prompting information filtering or automation support. Anticipatory physiological patterns, such as changes in HRV or electrodermal activity prior to task transitions, can signal reduced projection capability, enabling early intervention before performance degradation occurs. Together, this operational framework bridges physiological sensing and real-time SA estimation, addressing key limitations of descriptive approaches and enabling integration with adaptive human–autonomy systems. This example illustrates how the proposed framework can be applied across operational domains such as surveillance, driving, and human–robot interaction, where continuous monitoring of operator state can support adaptive decision-making and improve system safety and performance.

### 5.6 Summary of contributions

This section moves beyond descriptive aggregation of prior work by introducing a structured and operational framework on physiological situation-awareness (SA) estimation. First, it defines measurable physiological indicators associated with each SA level, grounded in observable features and temporal characteristics. Second, it proposes an operational framework that links these indicators to decision logic and probabilistic inference, explicitly accounting for real-time constraints and signal selection. Third, it introduces a hierarchical and predictive model of SA dynamics, in which perception, comprehension, and projection are modeled as a sequential process supported by multimodal physiological evidence. Together, these contributions provide a coherent and testable framework that bridges physiological sensing, cognitive modeling, and real-time human–autonomy interaction, thereby addressing key limitations of prior literature that focused primarily on modality-level descriptions without operational integration.

## 6 Physiological sensing modalities and their contributions to situation-awareness

**Figure 3:**
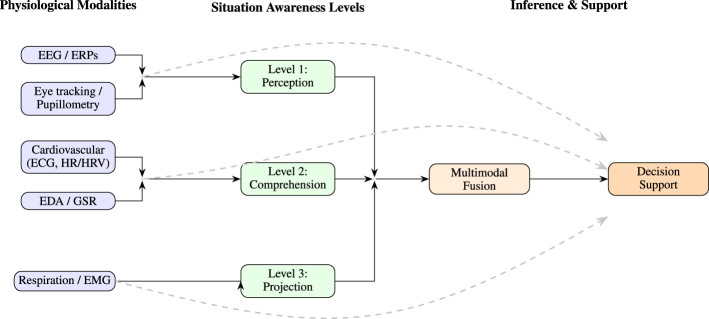
Physiological modalities aligned with the situation-awareness (SA) levels they most strongly inform. The diagram illustrates how representative sensing channels contribute to perception, comprehension, and projection processes within the broader physiological SA estimation pipeline introduced in Figure 1. Solid arrows represent hierarchical information flow toward multimodal fusion and decision-support mechanisms, while dashed arcs indicate indirect or contextual influence of physiological signals on higher-level SA inference.

In this section, physiological sensing modalities are interpreted through the lens of the perception–comprehension–projection framework in order to clarify how observable physiological signals relate to the underlying cognitive mechanisms that constitute situation awareness. Building on the evidence summarized in Section 3 and the multimodal integration strategies discussed in Section 4, this section synthesizes how physiological sensing modalities provide signals that can be used to estimate situation awareness (SA) during task execution. Each modality captures complementary aspects of the cognitive processes supporting the three canonical levels of SA: **perception** (detecting and attending to cues), **comprehension** (integrating information to form meaning), and **projection** (anticipating future states). However, physiological signals rarely correspond exclusively to a single SA level. For example, EEG markers such as the P300 reflect perceptual updating, whereas oscillatory indices such as mid-frontal theta correlate with working memory, cognitive control, and anticipatory processing (Polich, 2007; Cavanagh and Frank, 2014). Consequently, physiological modalities should be interpreted as providing overlapping and complementary indicators of SA processes rather than isolated measurements of individual SA levels. Figure 3 provides a conceptual overview of how representative physiological sensing channels map onto the perception, comprehension, and projection levels of SA and how these signals can be integrated through multimodal inference mechanisms to support decision-making. The following subsections summarize the principal physiological sensing modalities used for SA assessment, highlighting their underlying mechanisms, their relevance to the three levels of situation awareness, and their methodological strengths and limitations.

### 6.1 Electroencephalography/event-related potentials

Electroencephalography measures scalp-recorded neural activity and provides a direct window into brain processes associated with perception, attention, and cognitive control. Common EEG metrics used in SA research include event-related potentials (ERPs), spectral power in alpha and theta frequency bands, and frontal midline theta activity. For example, the P300 component represents a positive deflection in the EEG signal that occurs approximately 250–500 ms after a stimulus and is commonly associated with stimulus detection and attentional allocation. In the context of situation awareness, EEG markers relate to multiple SA levels. P300 or P3b amplitudes reflect perceptual detection and attentional capture, alpha suppression and frontal theta are often associated with working-memory load and cognitive integration, and sustained frontal theta has been linked to proactive monitoring and anticipatory control processes. EEG offers very high temporal resolution and direct access to neural processes underlying SA, although it is sensitive to movement artifacts and often requires careful preprocessing and within-subject calibration. When combined with complementary modalities such as eye tracking or autonomic signals, EEG can contribute to richer multimodal representations of cognitive state during complex tasks.

### 6.2 Eye tracking and pupillometry

Eye tracking and pupillometry provide behavioral and physiological indicators of visual attention and cognitive effort. Typical metrics include fixation duration, saccade patterns, scanpath structure, and changes in pupil diameter. These signals provide insight into different components of situation awareness. Fixation probability and time-to-first-fixation indicate where attention is allocated and therefore provide indicators of perceptual-level SA. The structure of scanpaths reflects how spatial and contextual cues are integrated during comprehension of the environment. In addition, pupil dilation is commonly associated with cognitive effort and increases during anticipatory preparation for upcoming task demands. Eye tracking provides a direct measure of overt visual attention and has demonstrated strong correlations with SA performance in many operational domains. However, it primarily reflects overt rather than covert attentional processes. In multimodal SA estimation frameworks, ocular metrics are often integrated with neural or autonomic signals to improve robustness and contextual interpretation.

### 6.3 Cardiovascular signals (ECG/PPG, HR, HRV)

Cardiovascular measures provide information about autonomic nervous system responses associated with workload, stress, and vigilance. These signals are typically derived from electrocardiography (ECG) or photoplethysmography (PPG), from which heart rate (HR), inter-beat intervals, and heart-rate variability (HRV) can be computed in both time and frequency domains. Compared with neural or ocular measures, cardiovascular signals exhibit slower temporal dynamics. Consequently, they show limited sensitivity to rapid perceptual detection processes but provide useful indicators of sustained cognitive effort and workload during information integration. Variations in HR and HRV have also been associated with vigilance fluctuations and anticipation of future task events. Cardiovascular signals are relatively easy to collect in operational environments and are robust to moderate movement. However, because they reflect aggregated autonomic activity, their interpretation can be influenced by physical state, emotional factors, and environmental conditions. For this reason, cardiovascular signals are typically interpreted in combination with faster neural or behavioral indicators within multimodal fusion frameworks.

### 6.4 Electrodermal activity/galvanic skin response

Electrodermal activity measures changes in skin conductance associated with sympathetic nervous system activation. Two primary components are typically analyzed: tonic skin conductance level (SCL), which reflects slower variations in baseline arousal, and phasic skin conductance responses (SCRs), which capture transient reactions to salient stimuli. In the context of situation awareness, phasic SCRs are often interpreted as orienting responses to surprising or unexpected environmental cues and may therefore relate to perceptual-level SA. Tonic arousal levels provide indicators of sustained engagement and cognitive–emotional workload during situation comprehension. Additionally, anticipatory SCRs may occur before expected critical events, suggesting a relationship with projection and anticipatory processes. EDA is highly sensitive to emotional and attentional states, although its signals can be affected by factors such as stress, thermoregulation, and environmental conditions. Within multimodal SA monitoring systems, electrodermal signals often complement neural and ocular measures by providing additional information about arousal and affective engagement.

### 6.5 Respiration, EMG, and other peripheral channels

Additional peripheral physiological signals, including respiration and electromyography (EMG), can provide complementary information about cognitive and affective states during task performance. Respiratory signals capture changes in breathing rate, variability, and thoracic expansion, which often shift with sustained workload and cognitive effort. Facial EMG activity can reflect subtle affective responses to stimuli and may provide indicators of momentary reactions to environmental cues. In some vigilance tasks, respiratory deceleration or rhythmic adjustments have been observed to precede planned responses, suggesting a possible association with anticipatory processes. Although these peripheral signals individually offer relatively low specificity for estimating situation awareness, they can provide useful contextual information when integrated with neural, ocular, and autonomic signals in multimodal physiological models.

### 6.6 Cross-modal synthesis of physiological indicators of situation awareness

Together, these modalities span multiple time scales and cognitive sub-processes involved in SA. To provide a structured and analytical overview of these relationships, Table 1 summarizes how major physiological sensing modalities contribute to different components of situation awareness. In addition to mapping modalities to SA levels, the table introduces key analytical dimensions, including temporal scale, observability, interpretability, and real-time applicability. These dimensions enable systematic comparison across modalities and clarify their respective roles and constraints within operational SA estimation frameworks.

**Table 1:** Physiological modalities mapped to the three levels of situation awareness (SA), using the same color palette as Figure 3.

Modality	Timescale	Interpretability	Observability	Real-time use	Primary SA contribution		
					**Perception**	**Comprehension**	**Projection**
EEG / ERPs	Milliseconds	Medium (requires preprocessing; strong subject specificity)	Event-based (ERP), Derived (spectral features)	Medium (spectral), Low (ERP requires event alignment)	**P300—detection of salient stimuli** (Polich, 2007; Wickens, 2008)	Theta/alpha—cognitive load and integration (Klimesch, 1999)	Frontal theta—anticipatory control (Cavanagh and Frank, 2014)
Eye tracking/ pupillometry	ms (gaze); s (pupil)	High for gaze; medium for pupil (effort/arousal inference)	Direct (gaze), derived (scanpaths, pupil metrics)	High	**Fixation patterns—spatial attention allocation** (Holmqvist et al., 2011)	Scanpaths reveal integration of contextual features (Land, 2009)	Pupil dilation increases during anticipatory preparation (Mathôt, 2018)
Cardiovascular (ECG/PPG, HR, HRV)	Seconds to minutes (slow autonomic dynamics)	Medium (context-dependent; influenced by physical state)	Direct (HR), Derived (HRV metrics)	High (HR), Medium (HRV requires windowing)	Limited cue detection sensitivity (Mehler et al., 2012)	**HR/HRV track sustained workload and stress** (Mehler et al., 2012)	**HRV modulations reflect vigilance and anticipation** (Park and Thayer, 2014)
EDA/GSR	Seconds (phasic); minutes (tonic baseline)	Medium-low (sensitive to emotional and environmental factors)	Direct (SCL), event-based (SCR)	Medium	SCR peaks—orienting response to salient events (Sokolov, 1963)	Tonic arousal reflects ongoing engagement (Howells et al., 2010)	**Anticipatory SCRs may precede expected critical events** (Bach et al., 2010)
Respiration/EMG	Seconds (moderate response speed)	Medium (useful as contextual co-modality)	Direct (respiration rate, EMG amplitude)	High	EMG captures immediate affective reactions ('t Hart et al., 2018)	Respiratory rate increases with sustained cognitive load (Grassmann et al., 2016)	**Respiratory rhythm adjustments precede planned responses** (Miyamoto et al., 2022)

Colored cells highlight the SA level most strongly associated with each modality, while other cells indicate secondary or contextual contributions. Green = strongest Perception link; Yellow = strongest Comprehension; Orange = strongest Projection.

Color intensity highlights the SA level most strongly supported by each modality.

Beyond modality-level mechanisms, it is also important to examine how these signals have been operationalized in empirical studies. Table 2 therefore synthesizes representative experiments across multiple task domains, including visual search and vigilance, driving and navigation, operational simulation and virtual reality, and cognitive-control paradigms. This organization highlights how task demands influence the physiological signatures associated with different aspects of situation awareness.

**Table 2:** Representative studies linking physiological sensing to situation awareness (SA).

Study	Modality	SA measure	Lvl	Main finding
Visual search/vigilance				
(Wilson and Russell 2003b)—aircraft detection	EEG, ECG/HRV	SAGAT	L2–L3	Alpha suppression and HRV reduction tracked SA decline with time-on-task.
(Brookings et al. 1996)—radar detection	EEG	Performance	L1–L2	Frontal theta/beta covaried with workload and attentional engagement.
(Baldwin and Penaranda 2012)—friend/foe classification	EEG, eye tracking	SAGAT	L1–L2	Gaze dynamics correlated with EEG markers of perceptual updating.
(Singh et al. 2021)—UAV tracking	EEG, HRV	Behavioral logs	L1	HRV decline predicted attentional fatigue and reduced cue detection.
Driving/navigation				
(Riley et al. 2004)—simulated driving	EEG, ECG/HRV	SAGAT, SPAM	L2–L3	Theta increase and HRV reduction indicated overload and reduced projection.
(Li et al. 2025)—maritime/navigation	EEG, HRV, EDA	SAGAT	L3	SA degradation under uncertainty reflected in joint EEG–autonomic signatures.
(Brookhuis and de Waard 2010)—on-road driving	HRV, EDA	Self-report	L2–L3	Autonomic changes reflected sustained load and reduced SA.
(Heikoop et al. 2018)—automated driving	EEG, eye tracking	SAGAT	L1–L2	Occipital alpha and blink metrics indicated attentional disengagement.
(Zhang et al. 2022)—shared control	EEG, EDA	Performance	L2–L3	Multimodal fusion improved SA-related prediction over single modalities.
Operational / simulation / VR				
(Dorneich et al. 2012)—C2 simulation	EEG, HRV	SAGAT	L2–L3	SA decline coincided with increased workload and reduced HRV.
(Qian et al. 2024b)—aviation simulation	EEG, ECG, eye tracking	SAGAT	L1–L2	Fusion outperformed single modalities in tracking perception/comprehension.
Aric et al. (2016)—ATC automation	EEG, ECG	Objective+subjective	L2–L3	EEG-driven adaptive automation improved SA maintenance.
(McMahan et al. 2015)—VR shooter	EEG, EDA	Performance	L1–L2	Arousal signals did not necessarily imply higher SA.
Verdière et al. (2018)—UAV teleop	EEG, eye tracking	SPAM	L1–L3	Combined EEG–gaze features best predicted SA decay.
Cognitive control/working memory				
(Parasuraman et al. 2009)—n-back	EEG, HRV	Performance/workload	L2	Frontal theta and HRV decrease mirrored workload-driven SA fluctuations.
(Matthews et al. 2015)—dual-task	EEG, EDA	SPAM	L2	Theta and EDA correlated with overload and reduced comprehension-level SA.
(Jung et al. 2018)—time pressure	EEG, ECG	SAGAT	L3	Stress-related EEG/HR changes predicted poorer projection accuracy.
(Lanata et al. 2021)—uncertainty	ECG, EDA	Self-report	L2–L3	Sympathetic activation predicted misjudgment under uncertainty.

Lvl: L1 = perception, L2 = comprehension, L3 = projection. SPAM, Situation Present Assessment Method; SAGAT, Situation Awareness Global Assessment Technique.

Importantly, the analytical dimensions highlighted in Table 1 reveal systematic differences in how physiological signals can be used for SA estimation. In particular, fast and directly observable signals (e.g., gaze and EEG spectral features) are more suitable for real-time estimation of perception-level SA, whereas slower or derived signals (e.g., HRV or electrodermal responses) provide more robust indicators of sustained cognitive integration and anticipation but require longer temporal windows and contextual interpretation. Across the studies summarized in Table 2, several methodological differences emerge. First, task paradigms vary considerably, ranging from controlled visual search experiments to complex operational simulations and navigation tasks. Second, sample sizes remain relatively small in most studies, often below 30 participants, which limits statistical power and constrains the application of data-intensive machine-learning approaches. Third, physiological modalities differ substantially in temporal resolution, observability, and robustness to noise, directly impacting their suitability for real-time SA estimation. These differences partly explain the variability in reported findings and highlight the need for more standardized experimental protocols and evaluation frameworks.

Two consistent patterns emerge in these paradigms. First, most studies rely on multimodal physiological sensing because individual physiological channels capture only partial aspects of operator awareness. Second, relatively few studies explicitly disentangle how individual physiological modalities map onto specific SA levels. These observations underscore the importance of interpreting physiological signals within a structured, task-dependent framework. The same physiological marker, for example, heart-rate variability, alpha suppression, or pupil dilation, may reflect distinct cognitive or affective processes depending on operational demands. Consequently, physiological modalities should not contribute equally to a general estimate of SA. Instead, their influence should be weighted according to (1) their theoretical relationship to specific SA levels, (2) their temporal dynamics and observability, and (3) their real-time applicability within the task context. Such task-aware weighting provides a principled foundation for developing multimodal SA metrics that are both interpretable and operationally meaningful. This structured view moves beyond a purely descriptive synthesis by identifying common dimensions across modalities and providing a foundation for operational and computational SA modeling. Recent studies further reinforce these patterns, demonstrating that multimodal physiological fusion improves sensitivity to SA degradation in aviation (Qian et al., 2024b), navigation (Li et al., 2025), and ergonomics-driven adaptive interface contexts (Smith et al., 2025).

## 7 Ethics, privacy, and data security

Physiological data collection in human–autonomy teaming (HAT) environments introduces not only ethical and privacy obligations but also mission-critical security considerations. Because autonomous agents, wearable sensors, and networked decision-support systems form a shared cyber–physical ecosystem, adversarial actors who compromise any component of this ecosystem could gain access to sensitive physiological data from military operators. Such data embed information about fatigue, stress, vigilance, and cognitive readiness—signals that, if intercepted or manipulated, could undermine operational integrity or even pose direct risks to personnel. Accordingly, approaches for safeguarding multimodal physiological data must be designed with both privacy protection and adversarial resilience in mind.

Acquisition and analysis of physiological data, particularly multimodal recordings that integrate neural, ocular, and autonomic signals, introduce complex ethical, legal, and privacy challenges. These data streams can expose sensitive aspects of cognition and emotion that extend beyond task performance, such as stress, fatigue, or affective state, thus increasing the risk of unintended inference or misuse. Responsible research in this domain requires strict adherence to ethical guidelines and transparent governance at every stage of the data lifecycle. Informed consent must explicitly describe the nature of the collected physiological signals, the possible inferences that may be drawn from them, and the confidentiality limits. Data should be pseudonymized and stored on encrypted access-controlled servers with verifiable audit trails.

When wireless acquisition systems or cloud-based analytics are used, secure transmission standards, such as TLS or WPA3, must be implemented to prevent interception, spoofing, or tampering. Software frameworks that manage synchronized physiological streams, such as the Lab Streaming Layer, should comply with institutional cybersecurity policies and maintain strict version control for reproducibility. For HAT systems specifically, safeguarding the communication channels between the operator and autonomous agent is essential; adversarial attacks that manipulate physiological telemetry could distort SA estimates or influence the autonomy's behavior in unpredictable ways.

Operational or defense applications require heightened ethical vigilance. In such contexts, physiological monitoring may intersect with decision-making authority, privacy rights, and the ethics of surveillance. Because deep-learning models trained on physiological data may influence decisions related to situational assessment, trust calibration, or even engagement criteria, their design and deployment must remain transparent, auditable, and open to human oversight. The principle of the human-in-the-loop is essential: automated situation-awareness systems should augment, not replace, accountable human judgment.

## 8 Limitations and open research questions

Despite significant methodological progress, several limitations constrain the current state of physiological situation-awareness research. Data scarcity remains a fundamental obstacle. Most published datasets include only a few dozen participants, short recording durations, and limited diversity in task types or environmental conditions. The resulting models are often overfitted to specific experimental paradigms and fail to generalize across contexts. Moreover, ground-truth estimation continues to challenge the field. Probes such as SAGAT or SPAM provide sparse, discontinuous labels that are difficult to align with continuous physiological signals, hindering the training and validation of temporal models. This misalignment underscores the need for semi-supervised and weakly supervised learning frameworks capable of leveraging unlabeled data while preserving interpretability. Inter-individual variability further complicates generalization. Physiological responses to workload, vigilance, and stress vary widely between operators, equipment configurations, and mission phases. Domain-adaptation and transfer-learning techniques could help mitigate these discrepancies, but their potential has yet to be systematically explored for situation-awareness modeling. Model transparency presents an equally critical challenge. Deep architectures, though powerful, remain largely opaque, making it difficult to relate latent features to interpretable physiological mechanisms. Incorporating attention-based visualization, gradient attribution, or physics-informed constraints may help illuminate how learned representations correspond to cognitive processes. A final limitation concerns ecological validity. Many experimental protocols rely on simplified laboratory simulations that do not fully capture the uncertainty, multitasking, and environmental variability of real-world operations. Expanding the evaluation to field conditions, where noise, movement, and dynamic teaming are prevalent, is essential to ensure robustness and operational relevance. In addition, most studies focus on individual operators rather than distributed teams. Modeling of shared and collective SA in human–autonomy teaming remains largely uncharted territory, demanding new frameworks that link multimodal physiology with communication dynamics, trust, and coordination behavior.

## 9 Conclusions and recommendations

This review has traced the evolution of physiological situation-awareness estimation from single-modality, handcrafted approaches toward integrated, multimodal, and representation-learning frameworks. By combining EEG, ocular, and autonomic signals within deep and self-supervised architectures, researchers have begun to capture the complementary dynamics of perception, comprehension, and projection that define the cognitive structure of SA. These developments suggest a paradigm shift toward continuous, unobtrusive, and adaptive estimation capable of supporting decision-making in both visual-search tasks and human–autonomy teaming (HAT). Future progress will depend on several convergent efforts. Multimodal synchronization must become standard practice, ensuring that neural, ocular, and autonomic data are temporally aligned with behavioral and contextual indicators. Label-efficient learning, particularly self-supervised pre-training on large physiological corpora followed by targeted fine-tuning, offers a scalable path to overcoming the limitations of small, labeled datasets. Equally important is embedding interpretability directly within model architectures. Attention mechanisms, physics-informed constraints, and *post-hoc* visualization tools can make learned representations transparent and physiologically meaningful, fostering trust among operators and human-factors researchers alike. Ethical and privacy considerations must evolve in parallel with technical innovation. Transparent documentation of data provenance, algorithmic decision rules, and model updates is critical to maintaining accountability, especially in safety-critical or defense contexts. Finally, advancing from controlled laboratory tasks to ecologically valid real-time environments will determine whether these models can truly function as adaptive collaborators rather than passive observers.

In summary, multimodal physiological sensing, when coupled with principled machine-learning design and rigorous ethical stewardship, offers a transformative avenue for understanding and enhancing human SA in the age of intelligent and autonomous systems. Beyond summarizing sensing modalities, this review emphasizes that physiological SA estimation should be understood as a cognitive modeling problem in which physiological signals serve as observable indicators of perceptual, integrative, and anticipatory processes underlying situation awareness.
